# Supplementary material for: Transfer Learning Meets Embedded Correlated Wavefunction Theory for Chemically Accurate Molecular Simulations: Application to Calcium Carbonate Ion Pairing
Source: J Chem Theory Comput. 2026 May 7;22(10):5174–84. doi: 10.1021/acs.jctc.6c00403 (PMC13217543; doi:10.1021/acs.jctc.6c00403)
Supplement: Supplementary file 1 [file ct6c00403_si_001.pdf]

Supporting Information: Transfer Learning Meets Embedded Correlated Wavefunction Theory for Chemically Accurate Molecular Simulations: Application to Calcium Carbonate Ion-Pairing

Xuezhi Bian[1] and Emily A. Carter[2,3]

[1]Department of Chemistry, Princeton University, Princeton, New Jersey 08544, United States

[2]Department of Mechanical and Aerospace Engineering, Princeton University, Princeton, New Jersey 08544, United States

[3]Andlinger Center for Energy and the Environment and Program in Applied and Computational Mathematics, Princeton University, Princeton, New Jersey 08544, United States

1. Details and Validation of Machine-Learned Interatomic Potentials
2. Comparison of Enhanced Sampling Methods
3. Temperature Effects
4. Details and Convergence Tests for Periodic ECW Calculations
5. Comparison Between ECW-TL and Direct Cluster Δ-Learning
6. Comparison of DFT-based FESs with previous related studies

1. Details and Validation of Machine-Learned Interatomic Potentials

The baseline DFT-revPBE-D3(BJ)-MLIP models were trained using a "training-exploration-labeling" active-learning workflow. The initial training dataset for the baseline model was selected from 24 5-ps constrained AIMD trajectories sampled at fixed Ca-C distances ranging from 2.6 Å to 5.4 Å (2.6, 2.8, 2.85, 2.9, 3.0, 3.05, 3.1, 3.2, 3.3, 3.4, 3.45, 3.5, 3.6, 3.7, 3.75, 3.8, 4.0, 4.2, 4.4, 4.6, 4.8, 5.0, 5.2, and 5.5 Å). 100 structures were sampled evenly along each trajectory, yielding a total of 2400 configurations. At each active-learning iteration, four MLIPs were trained independently with different random initial weights to form a committee. For the DP models, we employed the standard short-range, smooth two-body descriptor with a cutoff radius of $r_c = 6$ Å. The embedding network consisted of three layers with 25, 50, and 100 neurons, and 12 axis neurons, while the fitting network comprised three layers of 240 neurons each. Each model was trained for 400,000 steps using an exponentially decaying learning rate, which decreased from 10^{-3} to 10^{-8}. During training, we scheduled the energy prefactor in the loss function to increase from 0.02 to 1, while the force prefactor was set to decrease from 1000 to 1. These hyperparameters follow standard settings used for DP models trained for aqueous systems.

For the exploration stage, enhanced-sampling MD simulations based on the Ca-C distance were performed. Configurations exhibiting force deviations between 0.05 and 0.15 eV/Å among the 4 models were identified as uncertain and added to the training set. At each iteration, up to 500 configurations were selected and single-point DFT reference calculations were performed to obtain reference energies and forces, which were then added to the training dataset.

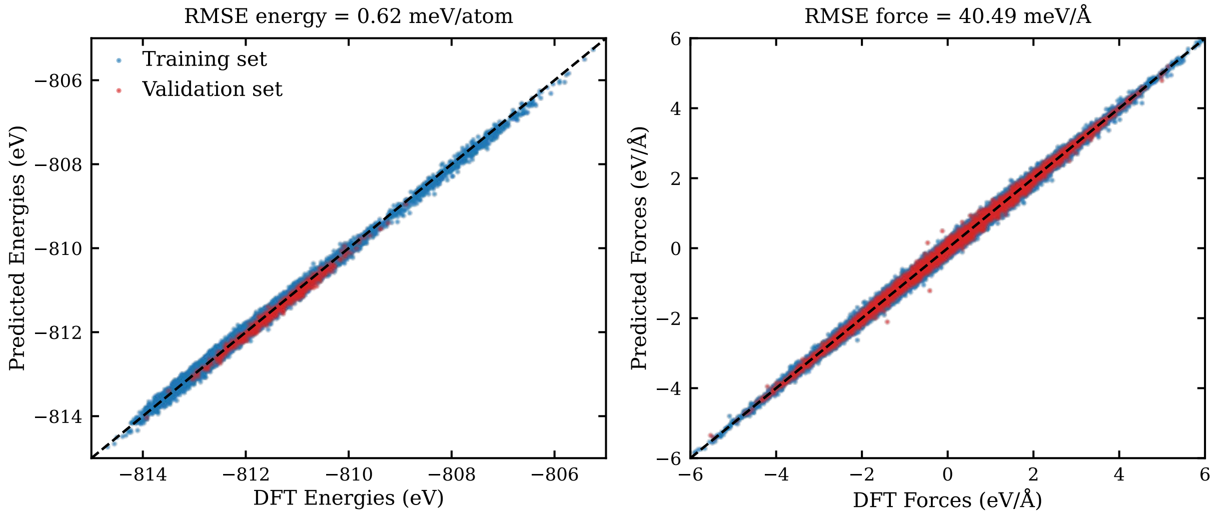

Figure S1 Comparison between DFT energies and forces and the baseline DFT-revPBE-D3(BJ) MLIP predicted energies and forces for both training and validation datasets. The model performs well on both datasets.

Active learning was considered converged after 12 iterations, because the difference between free-energy surfaces (FESs) computed in the last two active learning iterations was smaller than 0.2

kcal/mol across the entire sampled collective variable (CV) range, with a total of 6973 configurations accumulated. A final production model then was trained on this dataset for 1,000,000 ML steps to obtain the baseline DFT-revPBE-D3(BJ) model used in this work. The resulting model has a root-mean-square error (RMSE) of 0.62 meV/atom for energies and 40.49 meV/Å for forces on the training dataset. To validate the baseline model further, we performed 24 independent constrained MD simulations at the same fixed Ca-C distances as above for the initial training dataset, each 100 ps in length. 20 configurations were extracted from each trajectory at uniform time intervals, resulting in a validation set of 480 configurations. As shown in Fig. S1, the model exhibits errors in both energies and forces that are comparable to those observed in the training set.

Similarly, for the DFT-SCAN-MLIP model, the initial dataset of 500 configurations was randomly selected from the DFT-revPBE-D3(BJ) training set, and the same active-learning procedure was applied until convergence, resulting in a total of 7581 configurations. The final DFT-SCAN-MLIP model achieves an RMSE of 0.46 meV/atom for energies and 42.25 meV/Å for forces on the training dataset.

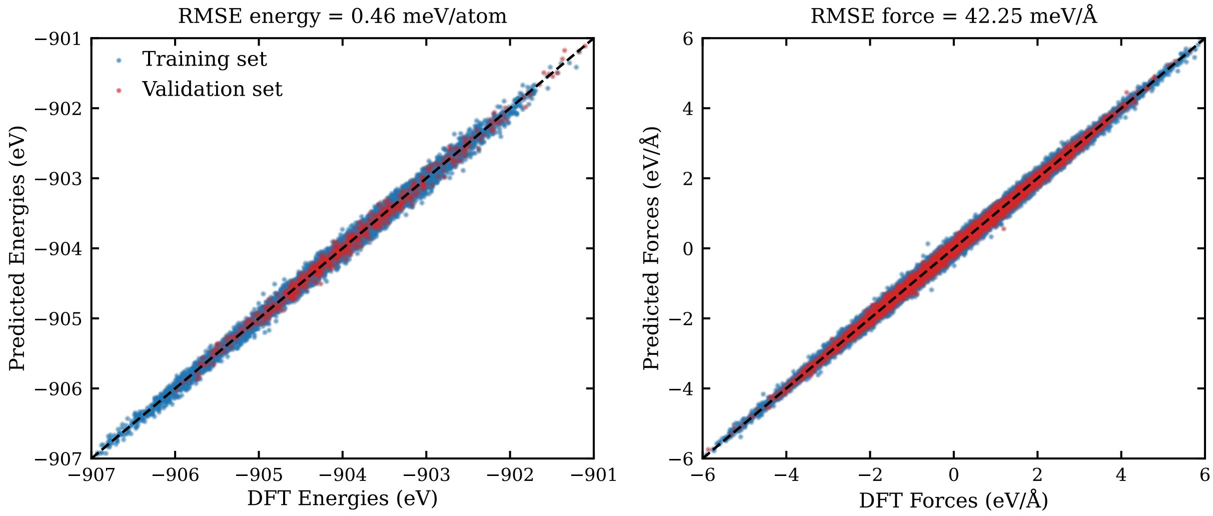

Figure S2 Same as Fig. S1, but for the DFT-SCAN-MLIP model. The model performs well on both training and validation datasets.

During transfer learning, the model was initialized from the baseline revPBE-D3(BJ) model. The embedding neural network was frozen, and we chose to apply a reduced learning rate starting from 10^{-4} decreasing to 10^{-8} over 400,000 steps. Because high-level ECW data contain only energies, we fixed the energy prefactor in the loss function to 1 and the force prefactor to 0. A larger learning rate or updating all parameters leads to catastrophic forgetting of the pretrained baseline model and results in unstable MD dynamics. Conversely, freezing too many parameters prevents the model from learning meaningful high-level corrections, causing it to behave similarly to the

baseline DFT model. The chosen empirical settings therefore represent a balance between retaining the baseline representation and incorporating the ECW-level information effectively.

2. Comparison of Enhanced Sampling Methods

For all FESs reported in the main text, we employed the on-the-fly probability enhanced sampling (OPES) method using a metadynamics-like target distribution,[1] in the LAMMPS[2] package with the PLUMED[3] plugin. During sampling, the bias potential was updated every 500 steps, with a maximum bias barrier of 50 kJ/mol. To prevent the Ca-C distance from sampling unphysically large values, we applied an upper wall at 6 Å with a harmonic force constant of 1000 kJ/mol/nm^2 which corresponds to approximately half the length of the simulation cell. All simulations were performed in the NVT ensemble using a canonical sampling with velocity-rescaling (CSVR) thermostat,[4] with a relaxation time of 100 ps. We used a 0.5 fs timestep, and we assigned a mass of 2 amu to hydrogen atoms for stable integration. This modification does not affect the resulting FES, which is an equilibrium observable independent of the particle masses.

For each FES, four independent trajectories of 2 ns were generated to ensure statistical reliability. The final free energy at a given $\boldsymbol{R}_{\mathrm{Ca-C}} = \boldsymbol{s}$ was calculated using standard reweighting of biased trajectories:

$$F(\boldsymbol{s}) = -\frac{1}{\beta}\ln\left(\frac{\langle \delta(\boldsymbol{R}_{\mathrm{Ca-C}} - \boldsymbol{s}) e^{\beta V(\boldsymbol{R})} \rangle}{\langle e^{\beta V(\boldsymbol{R})} \rangle}\right) \tag{S1}$$

where $\beta = 1/k_B T$, $V(\boldsymbol{R})$ is the bias potential at configuration $\boldsymbol{R}$, and $\langle \cdot \rangle$ represents the statistical average over the biased simulation.

Previous studies employing different enhanced-sampling techniques have reported noticeably different FES profiles even when using comparable electronic-structure methods. To assess the influence of the sampling method, we also computed the FES using the blue-moon ensemble (BME)[5,6] with our DFT-revPBE-D3(BJ) baseline MLIP model. 29 constrained trajectories of 400 ps each were propagated with the CP2K package,[7] with constraint values evenly spaced between $R_{\mathrm{Ca-C}} = 2.6$ to 5.4 Å. All BME simulations used the same CSVR thermostat parameters as the OPES simulations, with a 0.25 fs timestep and a hydrogen mass of 1 amu (because the mass cannot be set to 2 amu in CP2K to run these simulations, using a shorter timestep compensates for having to use the smaller mass). The free energy was then obtained from integrating the average constraint force according to

$$F(s) = \int_{s_0}^{s} ds \, \langle \lambda(s) \rangle - \frac{2}{\beta}\ln(s) \tag{S2}$$

where $\langle \lambda(s) \rangle$ is the statistical average of the Lagrange multiplier in the SHAKE algorithm[8] and the second term in Eq. (S2) is the entropy contribution from the Jacobi factor.

In Fig. S2, we compare the FES obtained from both the OPES and BME approaches. The two methods agree well, indicating that the ion-pairing FES is insensitive to the choice of enhanced sampling techniques. Nevertheless, both OPES and BME require relatively long sampling times to achieve convergence, which may help explain the discrepancies observed in some earlier AIMD-based studies.

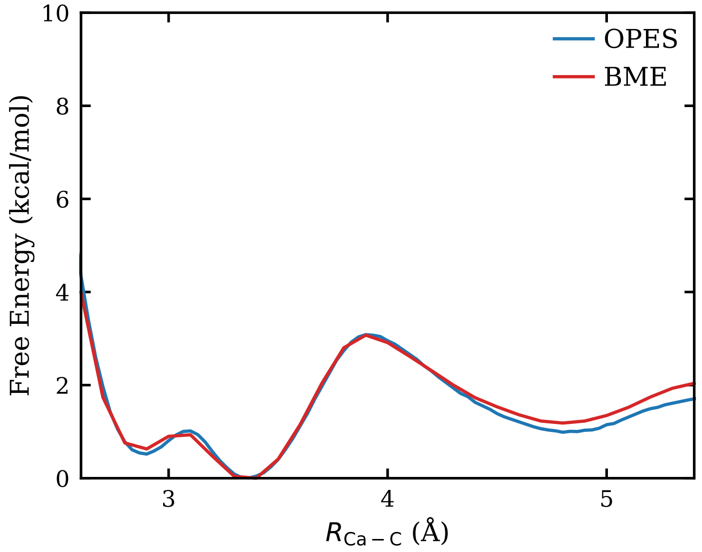

Figure S3 Free-energy surfaces computed using the BME and OPES enhanced-sampling methods. The two methods show excellent agreement.

3. Temperature Effects

Here we report the FES computed using the baseline DFT-revPBE-D3(BJ) MLIP model at different temperatures. As shown in Fig. S4, the temperature dependence in the bidentate and monodentate CIP regions is small, reflecting that the strong cation-ligand binding dominates the energetics in these configurations. In contrast, at the SSIP region and beyond, temperature effects become larger due to the increasing importance of entropy of the water configurations near and shared between the ion pair. Overall, the temperature dependence of the FES remains relatively small in the 300-330 K range. Looking forward, it will be interesting to explore the temperature dependence of the FES at higher temperatures using chemically accurate ECW-TL MLIP models to fully assess the role of entropic contributions.

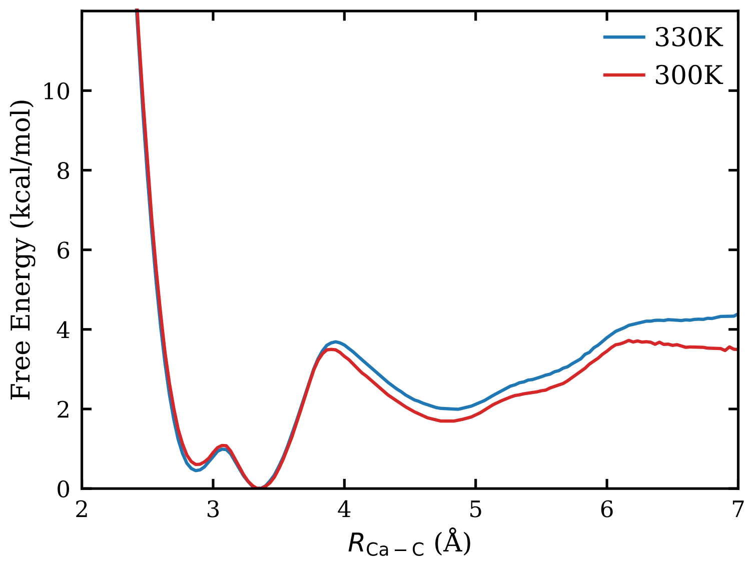

Figure S4 Free-energy surfaces computed at two temperatures with the DFT-revPBE-D3(BJ) MLIP model. The profiles show a relatively small temperature dependence at or near room-temperature.

4. Details and Convergence Tests for Periodic ECW Calculations

For the embedding potential generation, a 216 × 216 × 216 real-space grid is used for the fast Fourier transform (FFT) in the plane-wave DFT calculation, and the parameter F_{Corr} that controls the region of derivative corrections is set to 0.70. Within the projector augmented-wave (PAW) density functional embedding theory (DFET) method, the derivative correction is only evaluated for points within $F_{\text{Corr}}R_{\text{aug}}$, where R_{aug} is the core radius.[9] The embedding potential V_{emb} is optimized by maximizing the extended Wu-Yang functional to a gradient threshold of 10^{-5}. Once the embedding potential is optimized, we project it onto a Gaussian-type orbital (GTO) basis using the EmbeddingIntegralGenerator code[10] to perform ECW calculation:

$$V_{\mu\nu}^{\text{emb}} = \langle \mu | \hat{V}_{\text{emb}} | \nu \rangle \tag{S3}$$

where $V_{\mu\nu}^{\text{emb}}$ represents the one-electron embedding potential integral between atomic orbitals μ and ν. The ECW Hamiltonian can be expressed as

$$\hat{H}_{\text{ECW}} = \hat{H}_{\text{CW}} + \hat{V}_{\text{emb}}. \tag{S4}$$

where $\hat{H}_{\text{CW}}$ is the corresponding correlated wavefunction Hamiltonian of the cluster.

One issue with the current cell and cluster size is that the cluster sits too close to its periodic images. Although the embedding potential correctly accounts for interactions between the cluster and the environment - including all periodic replicas of the environment - it does not include the interactions between the cluster itself and its periodic images. To eliminate this artifact, we therefore used periodic GTO-based methods for the embedded DFT and ECW calculations with the ECW Hamiltonian defined in Eq. (S4), ensuring that the interactions between the cluster and both the periodic environment and cluster images are correctly included. We note this is the first time that the ECW framework has included these periodic images. Largely, the ECW method has been used to study metallic systems, which screen interactions rapidly, such that there was no need to include periodic images of the embedded clusters. But in lower dielectric constant condensed matter, it is fortunate that periodic CW methods and codes now can be utilized with ECW to properly account for all periodic interactions.

In PySCF,[11] the cell precision parameter controls the accuracy of periodic GTO integrals by setting the FFT grid, kinetic-energy cutoff, and lattice-summation cutoff. Figure S5 shows the convergence of SCF energies of periodic GTO DFT calculations with respect to the precision parameter. A precision value of 10^{-10} was used in this work, which yields convergence to better than approximately 0.001 kcal/mol or 1.6×10^{-6} Hartree.

For all periodic GTO calculations in this work, we used a large cc-pVTZ[12] basis set and a def2-TZVPP auxiliary basis[13] for density fitting to accelerate computations. We used a Gaussian smearing of 0.1 eV to stabilize SCF convergence and the SCF energies were converged to a threshold to 10^{-8} Hartree.

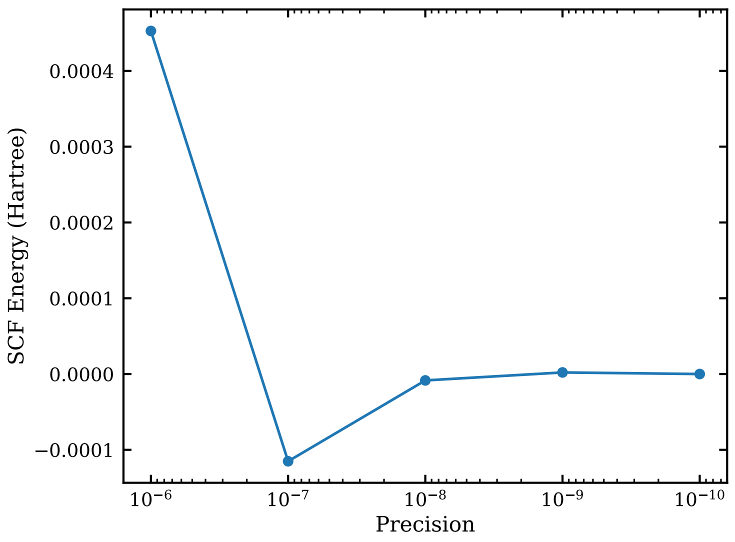

Figure S5 SCF energies as a function of the precision parameter in periodic GTO DFT calculations. A precision parameter of 10^{-10} provides good convergence. The energy zero is defined as the SCF energy calculated at 10^{-10}.

The embedded periodic GTO method was validated against the embedded periodic planewave (PW) method at the DFT-SCAN level (with periodic PW DFT-revPBE-D3(BJ) as the low-level theory for the total system). In Fig. S6, we present an energy parity plot comparing embedded periodic GTO DFT-SCAN and embedded periodic PW DFT-SCAN energies for the dataset used in the first fine-tuning iteration described in the main text (~700 configurations). The two methods agree quite well, considering these are total absolute energies (the error will be far less for actual energy observables, which are energy differences).

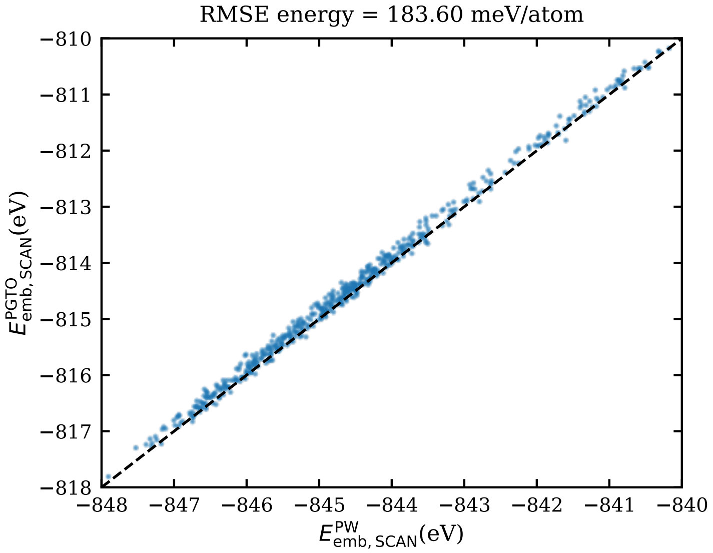

Figure S6 Comparison between embedded periodic GTO DFT-SCAN and embedded periodic PW DFT-SCAN energies, showing satisfactory agreement in total energies.

For the embedded periodic GTO MP2 and LNO-CCSD(T) calculations, after achieving convergence at the periodic Hartree-Fock level, we froze the inner-core 1s orbitals of C and O, and the 1s, 2s, and 2p orbitals of Ca for the later correlated calculations. For the embedded periodic LNO-CCSD(T) calculations,[14] first the canonical HF orbitals were localized using the Pipek-Mezey localization[15] to generate local orbitals and define fragments. The local subspace was defined by freezing occupied and virtual natural orbitals with eigenvalues below user-defined thresholds η_{occ} (η_{vir}) for each local fragments. The periodic LNO-CCSD(T) calculation then was carried out in the resulting truncated orbital space.[14] Note that at the $\eta_{\mathrm{occ}} = \eta_{\mathrm{vir}} = 0$ limit, LNO-CCSD(T) reduces to canonical CCSD(T). Here we fix the ratio $\eta_{\mathrm{occ}}/\eta_{\mathrm{vir}} = 10$ as recommended for molecular LNO-CC[16] and scan η_{vir} to analyze the convergence behavior.

The final embedded periodic LNO-CCSD(T) energies as a function of η_{vir} are plotted in Fig. S6.

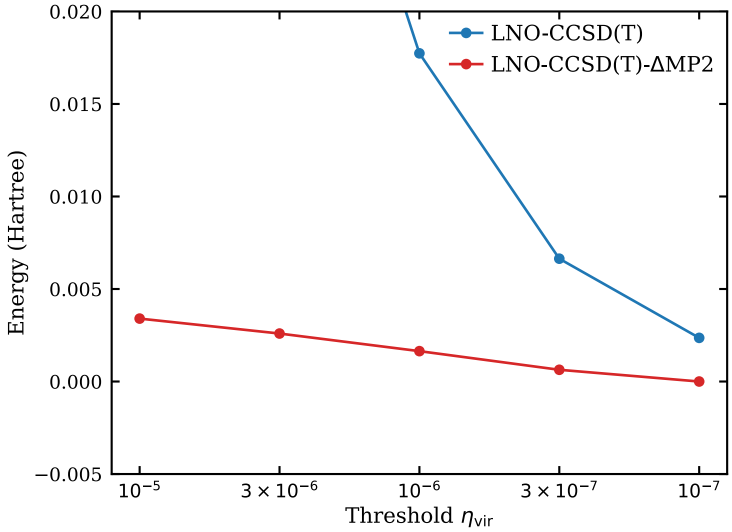

Figure S7 Embedded periodic LNO-CCSD(T) and periodic LNO-CCSD(T)-ΔMP2 energies as function of active space threshold η_{vir}. The energy zero is defined as the LNO-CCSD(T)- ΔMP2 energy calculated at $\eta_{\mathrm{vir}} = 10^{-7}$.

Although the direct LNO-CCSD(T) converges slowly with respect to the virtual space threshold, the MP2-corrected LNO-CCSD(T)-ΔMP2 defined as:

$$E_{\mathrm{LNO-CCSD(T)-\Delta MP2}} = E_{\mathrm{LNO-CCSD(T)}} + (E_{\mathrm{MP2}} - E_{\mathrm{LNO-MP2}}) \quad \text{(S5)}$$

converges to within 1 kcal/mol (or 1.6×10^{-3} Hartree) at $\eta_{\mathrm{vir}} = 10^{-6}$. Here, the MP2 correction term in parentheses in Eq. S5 represents the energy difference between periodic MP2 and periodic LNO-MP2. We therefore set $\eta_{\mathrm{vir}} = 10^{-6}$ as a compromise between computational cost and accuracy and use $E_{\mathrm{LNO-CCSD(T)-\Delta MP2}}$ as our reference energy in ECW-TL training.

5. Comparison Between ECW-TL and Direct Cluster Δ-Learning

Finally, we demonstrate the power of our ECW-TL framework by comparing it to the traditional Δ-learning approach. For the direct cluster Δ-learning approach, the high-level reference energy used to finetune baseline MLIP is defined as

$$E_{\Delta-\text{learning}} = E_{\text{baseline}} + \left(E_{\text{high-level}}^{\text{cluster}} - E_{\text{baseline}}^{\text{cluster}}\right) \tag{S6}$$

where the correction is constructed from an isolated cluster. The key difference between this Δ-learning approach and our ECW-TL framework is that it neglects all interactions between the selected cluster and the surrounding environment. We used the same configurations as in the transfer learning of our embedded-DFT-SCAN model in the main text, but with reference energies redefined according to Eq. (S6) to finetune the baseline DFT-revPBE-D3(BJ) model.

In Fig. S7, we plot the FES computed from both the embedded DFT-SCAN model and the direct cluster Δ-learning approach. Our embedded model clearly is more accurate than the isolated cluster model under the same transfer-learning setup. This result highlights the importance of properly accounting for system-environment interactions when fine-tuning MLIP models.

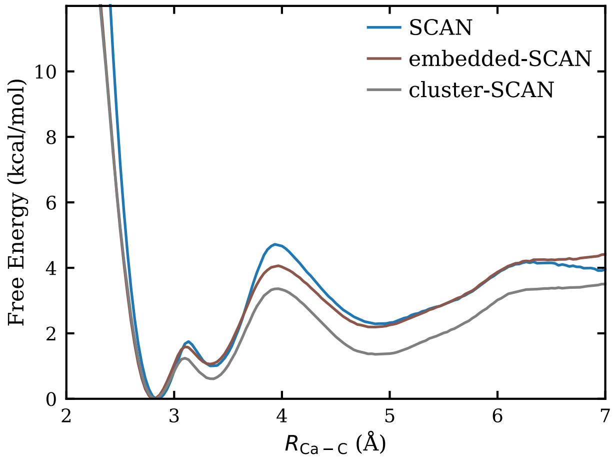

Figure S8 Free-energy surfaces computed from the two transfer-learning DFT-SCAN models compared with the DFT-SCAN reference model.

6. Comparison of DFT-based FESs with previous related studies

The FESs computed from our DFT-revPBE-D3(BJ) and DFT-SCAN models are compared in Fig. S9 with previous studies by Boyn et al.[17] and Piaggi et al. [18] . We find that the DFT-revPBE-D3(BJ)-MLIP-MD results in this work are in *qualitative* agreement with the DFT-revPBE-D3(BJ)-MD results of Boyn et al. in the bidentate and monodentate CIP regions. However, the DFT-MD results in Ref. 17 predict a significantly lower minimum in the SSHIP region, in disagreement with our results. These differences likely arise from limited sampling possible in the previous DFT-MD simulations, where each constrained MD trajectory was sampled for only 10 ps, which may not have been sufficient to achieve full convergence. This comparison highlights the advantage of MLIP-based simulations in obtaining well-converged FESs for ion-pairing systems.

For the MLIP-DFT-SCAN-based results, the model in this work and that of Ref. 18 again agree qualitatively. The remaining quantitative differences may arise from several factors. First, Piaggi et al. employed norm-conserving pseudopotentials, whereas we use all-electron, frozen-core PAW potentials. Second, their simulations were performed in the NPT ensemble, while ours are conducted in the NVT ensemble. Third, the model of Piaggi et al. was trained on a broader set of configurations spanning multiple processes, which may compromise accuracy for ion pairing compared to our model specifically trained for this system.

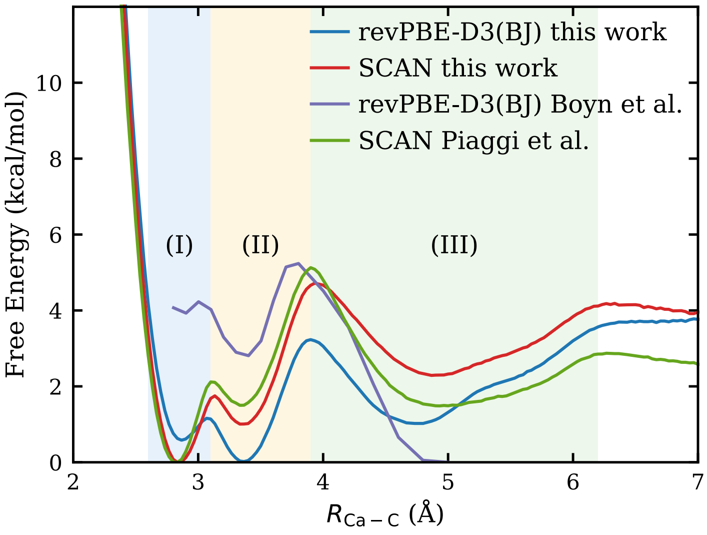

Figure S9 Free-energy surfaces computed from our two DFT-MLIP models compared with previous studies in Refs. 17 (revPBE-D3(BJ)-MD only) and 18 (SCAN-MLIP-MD only).

References

(1) Invernizzi, M.; Parrinello, M. Exploration vs Convergence Speed in Adaptive-Bias Enhanced Sampling. *J. Chem. Theory Comput.* **2022**, *18* (6), 3988–3996. https://doi.org/10.1021/acs.jctc.2c00152.

(2) Thompson, A. P.; Aktulga, H. M.; Berger, R.; Bolintineanu, D. S.; Brown, W. M.; Crozier, P. S.; in 't Veld, P. J.; Kohlmeyer, A.; Moore, S. G.; Nguyen, T. D.; Shan, R.; Stevens, M. J.; Tranchida, J.; Trott, C.; Plimpton, S. J. LAMMPS - a Flexible Simulation Tool for Particle-Based Materials Modeling at the Atomic, Meso, and Continuum Scales. *Comput. Phys. Commun.* **2022**, *271*, 108171. https://doi.org/10.1016/j.cpc.2021.108171.

(3) Tribello, G. A.; Bonomi, M.; Branduardi, D.; Camilloni, C.; Bussi, G. PLUMED 2: New Feathers for an Old Bird. *Comput. Phys. Commun.* **2014**, *185* (2), 604–613. https://doi.org/10.1016/j.cpc.2013.09.018.

(4) Bussi, G.; Donadio, D.; Parrinello, M. Canonical Sampling through Velocity Rescaling. *J. Chem. Phys.* **2007**, *126* (1), 014101. https://doi.org/10.1063/1.2408420.

(5) Carter, E. A.; Ciccotti, G.; Hynes, J. T.; Kapral, R. Constrained Reaction Coordinate Dynamics for the Simulation of Rare Events. *Chem. Phys. Lett.* **1989**, *156* (5), 472–477. https://doi.org/10.1016/S0009-2614(89)87314-2.

(6) DEN OTTER, W. K.; BRIELS, W. J. Free Energy from Molecular Dynamics with Multiple Constraints. *Mol. Phys.* **2000**, *98* (12), 773–781. https://doi.org/10.1080/00268970009483348.

(7) Kühne, T. D.; Iannuzzi, M.; Del Ben, M.; Rybkin, V. V.; Seewald, P.; Stein, F.; Laino, T.; Khaliullin, R. Z.; Schütt, O.; Schiffmann, F.; Golze, D.; Wilhelm, J.; Chulkov, S.; Bani-Hashemian, M. H.; Weber, V.; Borštnik, U.; Taillefumier, M.; Jakobovits, A. S.; Lazzaro, A.; Pabst, H.; Müller, T.; Schade, R.; Guidon, M.; Andermatt, S.; Holmberg, N.; Schenter, G. K.; Hehn, A.; Bussy, A.; Belleflamme, F.; Tabacchi, G.; Glöß, A.; Lass, M.; Bethune, I.; Mundy, C. J.; Plessl, C.; Watkins, M.; VandeVondele, J.; Krack, M.; Hutter, J. CP2K: An Electronic Structure and Molecular Dynamics Software Package - Quickstep: Efficient and Accurate Electronic Structure Calculations. *J. Chem. Phys.* **2020**, *152* (19), 194103. https://doi.org/10.1063/5.0007045.

(8) Ryckaert, J.-P.; Ciccotti, G.; Berendsen, H. J. C. Numerical Integration of the Cartesian Equations of Motion of a System with Constraints: Molecular Dynamics of *n*-Alkanes. *J. Comput. Phys.* **1977**, *23* (3), 327–341. https://doi.org/10.1016/0021-9991(77)90098-5.

(9) Yu, K.; Libisch, F.; Carter, E. A. Implementation of Density Functional Embedding Theory within the Projector-Augmented-Wave Method and Applications to Semiconductor Defect States. *J. Chem. Phys.* **2015**, *143* (10), 102806. https://doi.org/10.1063/1.4922260.

(10) Krauter, C. M.; Carter, E. A. EmbeddingIntegralGenerator. https://github.com/EACcodes/EmbeddingIntegralGenerator.

(11) Sun, Q.; Zhang, X.; Banerjee, S.; Bao, P.; Barbry, M.; Blunt, N. S.; Bogdanov, N. A.; Booth, G. H.; Chen, J.; Cui, Z.-H.; Eriksen, J. J.; Gao, Y.; Guo, S.; Hermann, J.; Hermes, M. R.; Koh, K.; Koval, P.; Lehtola, S.; Li, Z.; Liu, J.; Mardirossian, N.; McClain, J. D.; Motta, M.; Mussard, B.; Pham, H. Q.; Pulkin, A.; Purwanto, W.; Robinson, P. J.; Ronca, E.; Sayfutyarova, E. R.; Scheurer, M.; Schurkus, H. F.; Smith, J. E. T.; Sun, C.; Sun, S.-N.; Upadhyay, S.; Wagner, L. K.; Wang, X.; White, A.; Whitfield, J. D.; Williamson, M. J.; Wouters, S.; Yang, J.; Yu, J. M.; Zhu, T.; Berkelbach, T. C.; Sharma, S.; Sokolov, A. Yu.; Chan, G. K.-L. Recent Developments

in the PySCF Program Package. *J. Chem. Phys.* **2020**, *153* (2), 024109. https://doi.org/10.1063/5.0006074.
(12) Dunning, T. H. Gaussian Basis Sets for Use in Correlated Molecular Calculations. I. The Atoms Boron through Neon and Hydrogen. *J. Chem. Phys.* **1989**, *90* (2), 1007–1023. https://doi.org/10.1063/1.456153.
(13) Weigend, F.; Ahlrichs, R. Balanced Basis Sets of Split Valence, Triple Zeta Valence and Quadruple Zeta Valence Quality for H to Rn: Design and Assessment of Accuracy. *Phys. Chem. Chem. Phys.* **2005**, *7* (18), 3297–3305. https://doi.org/10.1039/B508541A.
(14) Ye, H.-Z.; Berkelbach, T. C. Periodic Local Coupled-Cluster Theory for Insulators and Metals. *J. Chem. Theory Comput.* **2024**, *20* (20), 8948–8959. https://doi.org/10.1021/acs.jctc.4c00936.
(15) Pipek, J.; Mezey, P. G. A Fast Intrinsic Localization Procedure Applicable for Ab Initio and Semiempirical Linear Combination of Atomic Orbital Wave Functions. *J. Chem. Phys.* **1989**, *90* (9), 4916–4926. https://doi.org/10.1063/1.456588.
(16) Rolik, Z.; Szegedy, L.; Ladjánszki, I.; Ladóczki, B.; Kállay, M. An Efficient Linear-Scaling CCSD(T) Method Based on Local Natural Orbitals. *J. Chem. Phys.* **2013**, *139* (9), 094105. https://doi.org/10.1063/1.4819401.
(17) Boyn, J.-N.; Carter, E. A. Characterizing the Mechanisms of Ca and Mg Carbonate Ion-Pair Formation with Multi-Level Molecular Dynamics/Quantum Mechanics Simulations. *J. Phys. Chem. B* **2023**, *127* (50), 10824–10832. https://doi.org/10.1021/acs.jpcb.3c05369.
(18) Piaggi, P. M.; Gale, J. D.; Raiteri, P. Ab Initio Machine-Learning Simulation of Calcium Carbonate from Aqueous Solutions to the Solid State. *Proc. Natl. Acad. Sci.* **2025**, *122* (41), e2415663122. https://doi.org/10.1073/pnas.2415663122.
